# A Correntropy-Based Proportionate Affine Projection Algorithm for Estimating Sparse Channels with Impulsive Noise

**DOI:** 10.3390/e21060555

**Published:** 2019-06-02

**Authors:** Zhengxiong Jiang, Yingsong Li, Xinqi Huang

**Affiliations:** 1 College of Information and Communications Engineering, Harbin Engineering University, Harbin 150001, China; 2 Key Laboratory of Microwave Remote Sensing, National Space Science Center, Chinese Academy of Sciences, Beijing 100190, China

**Keywords:** sparse channel estimation, maximum correntropy criterion, proportionate affine projection algorithm, impulsive noise environments

## Abstract

A novel robust proportionate affine projection (AP) algorithm is devised for estimating sparse channels, which often occur in network echo and wireless communication channels. The newly proposed algorithm is realized by using the maximum correntropy criterion (MCC) and the data reusing scheme used in AP to overcome the identification performance degradation of the traditional PAP algorithm in impulsive noise environments. The proposed algorithm is referred to as the proportionate affine projection maximum correntropy criterion (PAPMCC) algorithm, which is derived in the context of channel estimation framework. Many simulation results were obtained to verify that the PAPMCC algorithm is superior to early reported AP algorithms with different input signals under impulsive noise environments.

## 1. Introduction

A class of adaptive filtering (AF) algorithms are extensively considered in use in channel estimation (CE), echo cancellation, noise elimination, etc. [1,2,3,4,5,6,7,8,9,10]. For example, the well-known least mean square (LMS), normalized LMS (NLMS) and recursive least square (RLS) algorithms were used in various systems. Although the LMS algorithm has a simple principle and a small amount of computation in practice, it might converge slowly in low signal-to-noise ratio (SNR) scenes. In contrast, the RLS converges faster than the basic LMS. However, it is proved to have high cost of increased computational complexity, which will use more computing resources when the order of the AF is large. In addition, if the input signal is a speech signal, the convergence speed for the basic LMS algorithm becomes very slow as the eigenvalue distribution range for the input signal autocorrelation matrix is large [11]. To enhance the identification behaviors of the RLS and NLMS algorithms in practical engineering and to obtain high accuracy and fast convergence, the affine projection (AP) algorithm is proposed by reusing latest input signals to improve the NLMS’s performance [12]. The computation burden of the AP is between the LMS and RLS algorithms, and the AP algorithm has a fast convergence, especially for colored or speech signal input signals [13].

In many engineerings, such as speech signal processing and real-time traffic predictions, noise often exhibits strongly impulsive characteristics [14,15]. Traditional NLMS and AP algorithms, which use the minimum-mean-square-error (MMSE) criterion to construct an expected cost function, will suffer from performance degradation in those impulsive noise environments. To find the solution for handling these problems, the maximum correntropy criterion (MCC) and the minimum error entropy criterion (MEEC) have been proposed to give resistance to the impulsive noise [16,17]. Although the MEEC is a robust criterion, its computational complexity is very high, while the MCC algorithm whose computational complexity is comparable to the LMS has been widely used to resist the impulsive noise [18,19,20,21].

On the other hand, scholars found that the sparse characteristics are existing in a great number of scenarios such as network echo channels and underwater acoustic communication channels [22,23,24,25]. However, classical LMS, AP and MCC algorithms cannot take advantage of the sparse structures of these sparse channels. Then, the proportionate AF algorithms have been proposed to make use of the sparse information in the mentioned channels [26]. For example, the proportionate NLMS (PNLMS) combines the proportionate scheme into the NLMS to reassign the gains to each channel coefficients [26]. Then, proportionate-type AF algorithms were widely realized and utilized for channel estimation as well as the echo cancellation [27,28,29,30]. For the sake of comparison with the traditional NLMS, the PNLMS suffers from slow convergence if the input signal is driven by colored or speech signals, resulting in that steady-state error might be worse than that of the NLMS. Inspired by the PNLMS, the proportionate AP (PAP) algorithm has been proposed by using the idea in PNLMS to fully use the sparse structure-information of the echo channels [31] based on the data reusing principle. Then, various proportionate-type AP algorithms have been proposed [32,33,34,35,36,37]. However, the PAP-type algorithms have performance degradation in impulsive noise environments because of the MMSE scheme. Thus, the sign algorithms, such as affine projection sign (APS) algorithm and proportionate APS (PAPS) algorithm [38,39], are successfully used for dealing with impulsive noise. Additionally, another kind of sparse-aware APs have been reported and analyzed by taking the consideration of the compressed sensing (CS) theory [40]. With the help of the concept of the CS, a series of sparsity-aware AF algorithms, such as zero-attracting LMS (ZA-LMS), reweighted ZA-LMS (RZA-LMS), ZA-AP, and RZA-AP algorithms have been proposed within the AF [41,42,43,44,45,46,47].

In this paper, the AP scheme and MCC are considered together to construct a new cost function to enhance the PAP algorithm in impulsive noise environments, which is denoted as proportionate affine projection maximum correntropy criterion (PAPMCC) algorithm. The proposed PAPMCC algorithm is investigated by using α-stable distribution as the impulsive noise model. Experimental results verify that the PAPMCC provides a lower steady state error than AP, ZA-AP, RZA-AP, and PAP algorithms with different inputs.

## 2. Review of the PAP Algorithm

**Figure 1:**
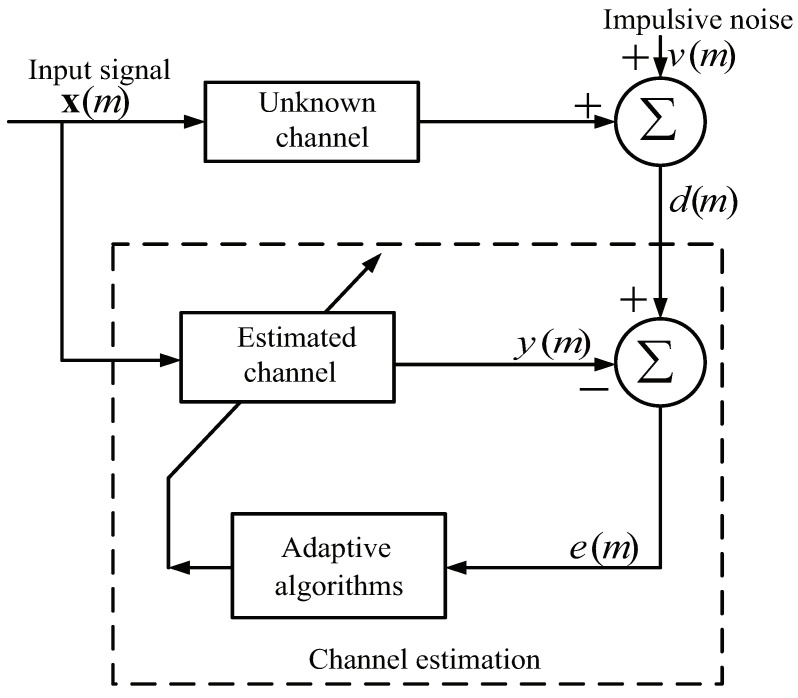
Typical CE schematic diagram.

In the range of AF, the implementation schematic diagram for CE is presented in Figure 1. Assume that the input signal x(m)=[x(m),x(m−1),⋯,$x\left(m-K+1\right){]}^{T}$ is used in this paper, and the channel impulse response (CIR) is modeled as $w\left(m\right)={\left[w_{0}\left(m\right),\cdots ,w_{K-1}\left(m\right)\right]}^{T}$, where *K* denotes as the total length and *m* denotes the time slot. Then, the received signal d(m) is

(1) $$d\left(m\right)=x^{T}\left(m\right)w\left(m\right)+r\left(m\right),$$

in which r(m) represents the additive impulsive noise that is usually independent of x(m), and ${}^{T}$ represents the transposed operation. The gotten CIR is given as $\hat{w}\left(m\right)={\left[{\hat{w}}_{0}\left(m\right),{\hat{w}}_{1}\left(m\right),\cdots ,{\hat{w}}_{K-1}\left(m\right)\right]}^{T}$, resulting in

(2) $$y\left(m\right)=x^{T}\left(m\right)\hat{w}\left(m\right).$$

The estimation error at *m* is expressed as

(3) e(m)=d(m)−y(m).

### 2.1. AP Algorithm

To the best of our knowledge, the AP algorithm reuses the current and previous input signal information, which achieves faster convergence compared with the NLMS when the input signal is colored. The input matrix for the AP algorithm is

(4) X(m)=[x(m),x(m−1),⋯,x(m−M+1)].

where *M* is a projection order. Due to the reuse of data, y(m) and the estimated error e(m) are expressed as

(5) $$y\left(m\right)=X^{T}\left(m\right)\hat{w}\left(m\right),$$

(6) $$d\left(m\right)={\left[d\left(m\right),d\left(m-1\right),\cdots ,d\left(m-M+1\right)\right]}^{T},$$

(7) $$\begin{array}{c} e(m)=d(m)-y(m). \end{array}$$

The iteration equation for the standard AP algorithm is given by

(8) $$\hat{w}\left(m+1\right)=\hat{w}\left(m\right)+\mu_{\mathrm{AP}}X\left(m\right){\left(X^{T}\left(m\right)X\left(m\right)+\delta_{\mathrm{AP}}I_{M}\right)}^{-1}e\left(m\right),$$

in which $\mu_{\mathrm{AP}}$ denotes the step size, $\delta_{\mathrm{AP}}>0$ is to prevent the matrix to be inverted to singular, and $I_{M}$ is a *M*-order identity matrix.

### 2.2. PAP Algorithm

From the inspiration of the well-known PNLMS algorithm, the PAP algorithm integrates the proportionate idea into the AP algorithm to modify the gain allocation method, and realizes a dynamic step size (STS) based on the magnitudes of the channel coefficients that are included in the unknown channels. The iteration equation of the PAP is modified to be

(9) $$\hat{w}\left(m+1\right)=\hat{w}\left(m\right)+\mu_{\mathrm{PAP}}G\left(m\right)X\left(m\right){\left(X^{T}\left(m\right)G\left(m\right)X\left(m\right)+\delta_{\mathrm{PAP}}I_{M}\right)}^{-1}e\left(m\right),$$

where $\mu_{\mathrm{PAP}}$ is still used as a STS, $\delta_{\mathrm{PAP}}$ denotes the regularization factor in the PAP, and G(m) acts as the gain controlling matrix, which is written as

(10) $$\begin{array}{c} G\left(m\right)=\mathrm{diag}\left\{\left.g_{0}\left(m\right),g_{1}\left(m\right),\ldots ,g_{K-1}\left(m\right)\right\}\right., \end{array}$$

where

(11) $$g_{k}\left(m\right)=\frac{\varphi_{k}\left(m\right)}{\sum_{i=0}^{K-1}\varphi_{i}\left(m\right)},$$

and

(12) $$\begin{array}{c} \varphi_{k}=\max\left\{p\max\left\{\left.q,\left|{\hat{w}}_{0}\right|,\left|{\hat{w}}_{k}\right|,\cdots ,\left|{\hat{w}}_{K-1}\right|\right\}\right.\right.,\left.\left|{\hat{w}}_{k}\right|\right\}. \end{array}$$

In Equation (12), parameters p>0 and q>0 are used to prevent the update process from stalling. In practice, $p=\frac{5}{K}$ is usually chosen [26].

## 3. Proposed PAPMCC Algorithm

The PAP can provide amazing convergence performance in Gaussian noise environments, but the performance will degrade under the impulsive noise environments. To take full use of the sparse characteristics of the CIRs, a robust PAP algorithm is realized by combining the proportionate idea with the basic AP and MCC together to construct the PAPMCC algorithm. As a result, the proposed PAPMCC algorithm solves the minimization problem given by

(13) $${∥\hat{w}\left(m+1\right)-\hat{w}\left(m\right)∥}_{G^{-1}\left(m\right)}^{2}\;\mathrm{subject}\;\mathrm{to}\;\overset{⌣}{\;e}\left(m\right)=\left[1_{M}-\xi \exp\left(-\frac{e(m)⊙e(m)}{2\sigma^{2}}\right)\right]⊙e\left(m\right),$$

where $\overset{⌣}{\;e}\left(m\right)=d\left(m\right)-X^{T}\left(m\right)\hat{w}\left(m+1\right)$, *σ* denotes the kernel width, and **1**_*M*_ is a column vector whose elements are ones. e(m)⊙e(m) denotes the Hadamard product between two estimated error vectors **e**(*m*). According to the Lagrange multiplier method (LMM) with multiple constraints, the cost function is presented as

(14) $$J\left(\hat{w}\left(m+1\right)\right)={∥\hat{w}\left(m+1\right)-\hat{w}\left(m\right)∥}_{G^{-1}\left(m\right)}^{2}+\lambda \left\{\overset{⌣}{\;e}\left(m\right)-\left[1_{M}-\xi \exp\left(-\frac{e(m)⊙e(m)}{2\sigma^{2}}\right)\right]⊙e\left(m\right)\right\},$$

where ***λ*** = [*λ*_1_, *λ*_2_, …, *λ_M_*]. Then, let

(15) $$\frac{\partial J(\hat{w}\left(m+1\right))}{\partial \hat{w}\left(m+1\right)}=0\;\mathrm{and}\;\frac{\partial J(\hat{w}\left(m+1\right))}{\partial \lambda }=0.$$

After performing algebraic operations, we get

(16) $$\hat{w}\left(m+1\right)=\hat{w}\left(m\right)+\frac{1}{2}G\left(m\right)X\left(m\right)\lambda^{T},$$

and

(17) $$d\left(m\right)=X^{T}\left(m\right)\hat{w}\left(m+1\right)+\left[1_{M}-\xi \exp\left(-\frac{e(m)⊙e(m)}{2\sigma^{2}}\right)\right]⊙e\left(m\right).$$

Solving Equations (16) and (17), the Lagrange multiplier vector is given by

(18) $$\lambda^{T}=2\xi {\left[X^{T}\left(m\right)G(m)X\left(m\right)\right]}^{-1}\exp\left(-\frac{e(m)⊙e(m)}{2\sigma^{2}}\right)⊙e\left(m\right).$$

Substituting Equation (18) into Equation (16), the iteration of the PAPMCC is expressed as

(19) $$\hat{w}\left(m+1\right)=\hat{w}\left(m\right)+\xi G\left(m\right)X\left(m\right){\left[X^{T}\left(m\right)G\left(m\right)X\left(m\right)\right]}^{-1}\exp\left(-\frac{e(m)⊙e(m)}{2\sigma^{2}}\right)⊙e\left(m\right).$$

In practice, Equation (19) can be corrected to

(20) $$\hat{w}\left(m+1\right)=\hat{w}\left(m\right)+\mu G\left(m\right)X\left(m\right){\left[X^{T}\left(m\right)G\left(m\right)X\left(m\right)+I_{M}\delta_{\mathrm{PAPMCC}}\right]}^{-1}\exp\left(-\frac{e(m)⊙e(m)}{2\sigma^{2}}\right)⊙e\left(m\right),$$

where μ=ξ acts as the step size, $\delta_{\mathrm{PAPMCC}}$ denotes the regularization factor, and G(m) is the weight assignment matrix that is defined in Equations (10)–(12).

The computation complexity of the devised PAPMCC algorithm is compared with the AP, ZA-AP, RZA-AP and PAP algorithms with respect to the total number of additions, multiplications, and divisions in each iteration. The comparison is presented in Table 1. It is clear to see that the computational complexity of the proposed PAPMCC algorithm is comparable to that of the PAP algorithm.

## 4. Experimental Results

**Figure 2:**
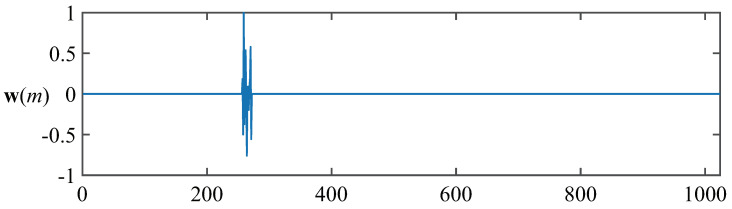
The impulse response used in simulation below.

Several experiments were constructed to give an analysis on the performance of the PAPMCC algorithm for implementing the sparse CE. Since the α-stable distribution can well construct the non-Gaussian phenomenon, which is ubiquitous in practice, it was chosen to model the impulsive noise in the simulations. The α-stable distribution function is defined as

(21) $$f\left(t\right)=\exp\left\{j\chi t-\gamma {\left|t\right|}^{\alpha }\left[1+j\beta \mathrm{sgn}(t)S(t,\alpha )\right]\right\},$$

where

(22) $$S\left(t,\alpha \right)=\left\{\begin{array}{c} \tan\frac{\alpha \pi }{2}\;\;\mathrm{if}\;\alpha \neq 1\\ \frac{2}{\pi }\log\left|t\right|\;\mathrm{if}\;\alpha =1, \end{array}\right.$$

in which α∈(0,2] represents the characteristic index, which controls the behavior of the impulsive distribution. When parameter α is smaller, the impulsive intensity becomes larger. β∈[−1,1] is the symmetric parameter, χ denotes positional parameter, and γ>0 represents the dispersion parameter. Furthermore, the α-stable distribution is given by $V_{\alpha -\mathrm{stable}}\left(\alpha ,\beta ,\gamma ,\chi \right)$. Herein, $V_{\alpha -\mathrm{stable}}\left(1.5,0,0.2,0\right)$ is chosen to implement the impulsive noise. In all simulation experiments, K=1024, and σ=1 were selected, and the input signal power was 1. The network echo channel used for the experiments, which is classical sparse channel presented in Figure 2, whose active coefficients distributed in [257,272], was considered to evaluate the proposed PAPMCC algorithm. The related parameters were set to be $\delta_{\mathrm{AP}}=\delta_{\mathrm{ZA}-\mathrm{AP}}=\delta_{\mathrm{RZA}-\mathrm{AP}}=0.01$ and $\delta_{\mathrm{PAP}}=\delta_{\mathrm{PAPMCC}}=\frac{1}{K}\delta_{\mathrm{AP}}$ [48]. The performance for all used algorithms was evaluated by normalized misalignment (NM), which is written as $10\log_{10}\left({∥w-\hat{w}∥}_{2}^{2}/{∥w∥}_{2}^{2}\right)$.

### 4.1. Performance of the PAPMCC Algorithm with Various Projection Orders M, Step-Sizes μ and Kernel Width σ

**Figure 3:**
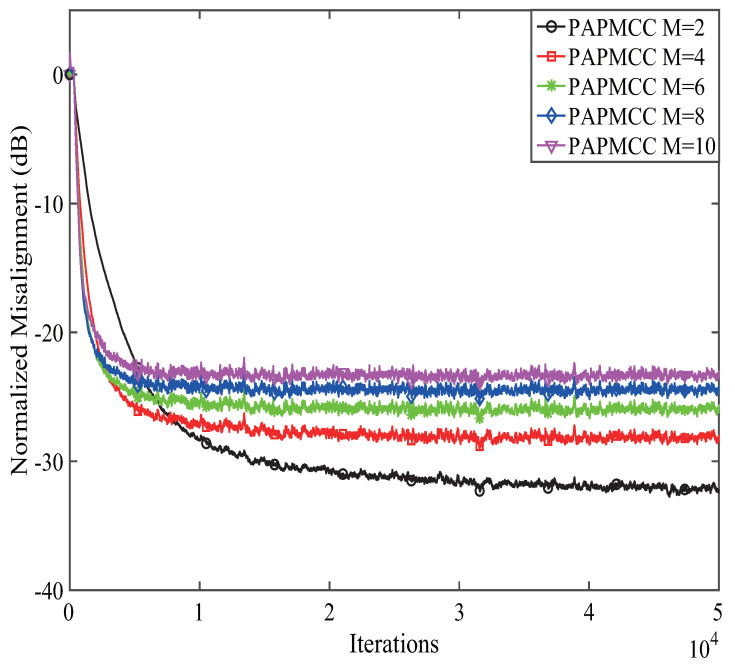
The effects of the projection orders on PAPMCC algorithm.

Firstly, the effects of the projection order *M* on the convergence for the PAPMCC algorithm was investigated. The colored noise, which was obtained from white Gaussian noise (WGN) filtering through an autoregressive with a pole at 0.8, was used as the input signal. Herein, μ=0.05. The results given in Figure 3 point out that increasing the projection order *M* could speed up the convergence, while the steady-state misalignment was increased. Therefore, a trade-off between the convergence speed and steady-state misalignment should be taken into consideration.

**Figure 4:**
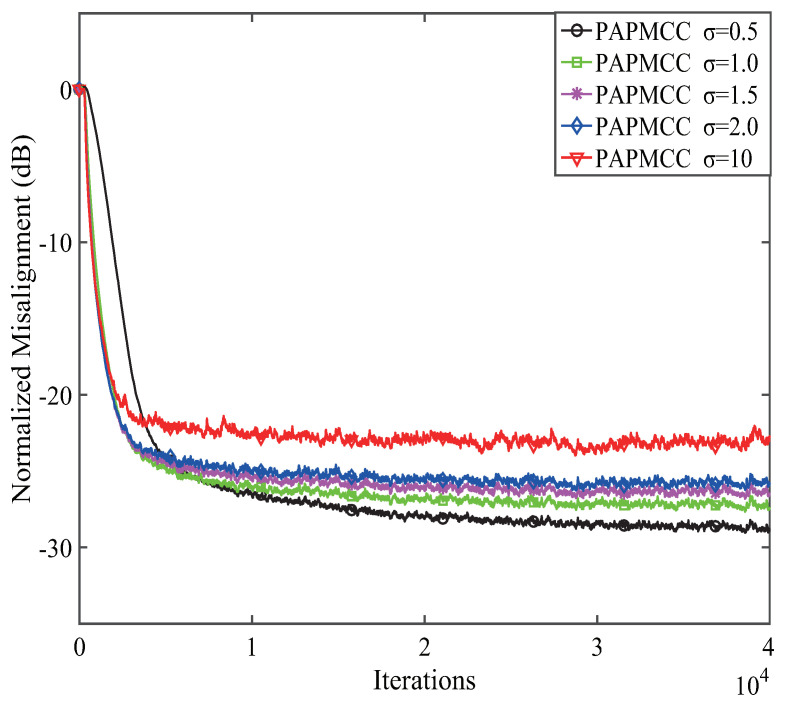
The effects of the kernel width σ on PAPMCC algorithm.

Secondly, the effects of kernel width σ on the convergence for the PAPMCC algorithm was analyzed and discussed. Herein, M=4 was selected. From Equation (20), the parameter σ affects the estimation behaviors of the PAPMCC algorithm, while σ is an important parameter for Gaussian kernel to suppress noise interference. Given the diversity and complexity of the target signal and noise, it is not easy to get the optimal solution of the kernel width σ from the theoretical derivation. Therefore, the simulation experiments were used to determine the appropriate value σ. The results given in Figure 4 point out that the steady-state error of the PAPMCC algorithm increased with the increment of σ. The PAPMCC algorithm had high estimation error when σ took a larger value since MCC behaved similar LMS when the value of σ was very large.

**Figure 5:**
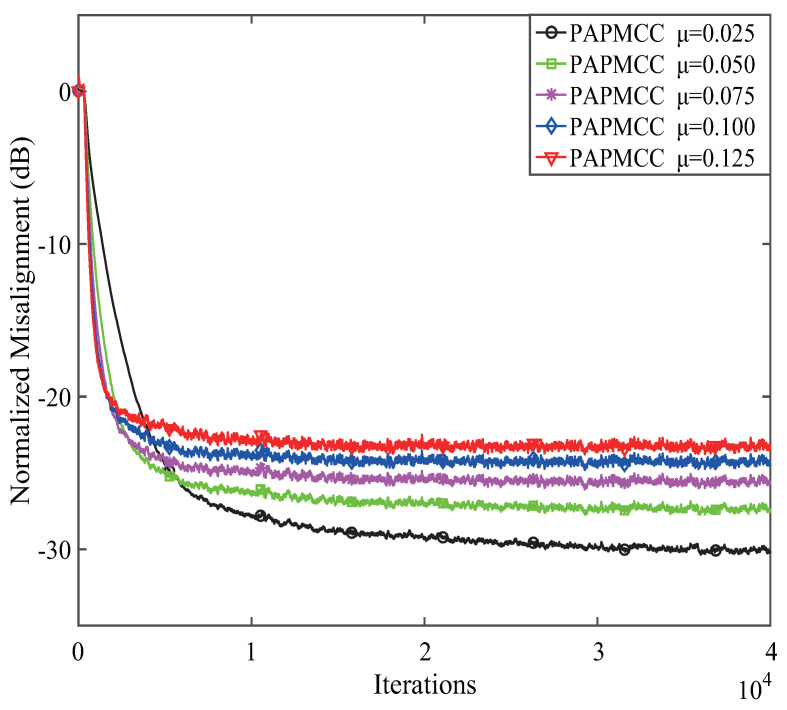
The effects of the step size μ on PAPMCC algorithm.

Thirdly, the effects of μ on the convergence for the PAPMCC algorithm was investigated using colored noise as the input signal. From the above simulation results, σ=1.0 was selected, and other parameters were the same as the first experiments, and the results are presented in Figure 5. Parameter μ controls the convergence speed of the PAPMCC. With the increment of μ, the normalized misalignment was decreased, while the convergence rate became fast. Consequently, the parameters μ and σ are supposed to be reasonably selected in practical application.

### 4.2. Performance Comparisons of the Proposed PAPMCC Algorithm under Different Input Signals

**Figure 6:**
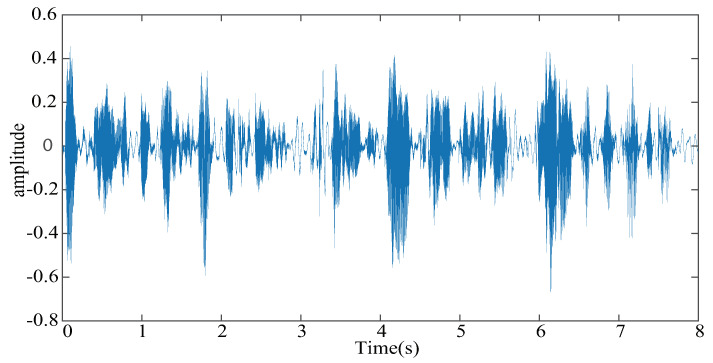
The actual speech signal which is used to estimate the network echo channel.

**Figure 7:**
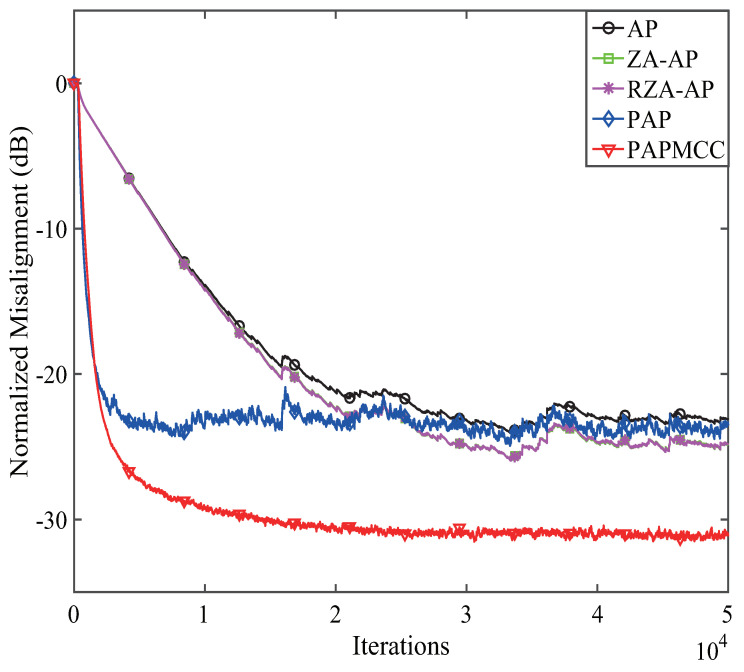
Performance comparisons of the proposed PAPMCC algorithm. Input signal: WGN.

**Figure 8:**
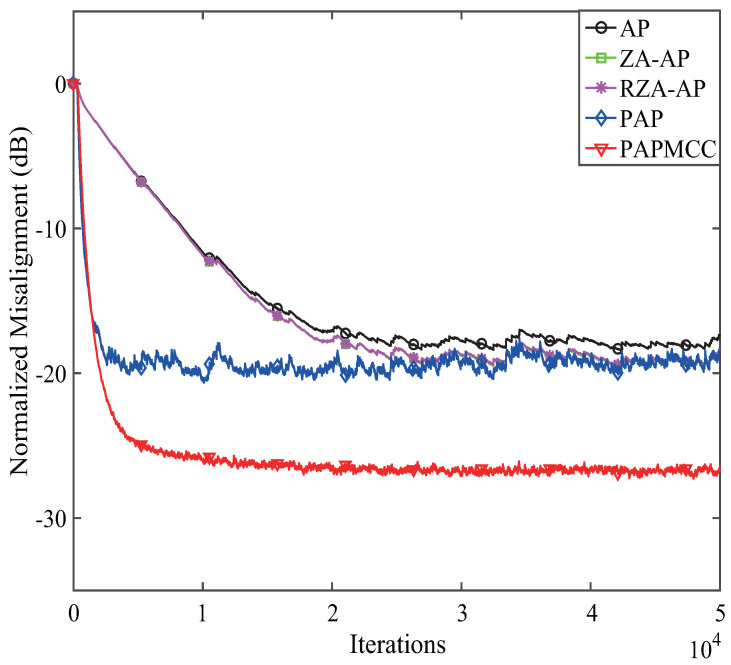
Performance comparisons of the proposed PAPMCC algorithm. Input signal: colored.

**Figure 9:**
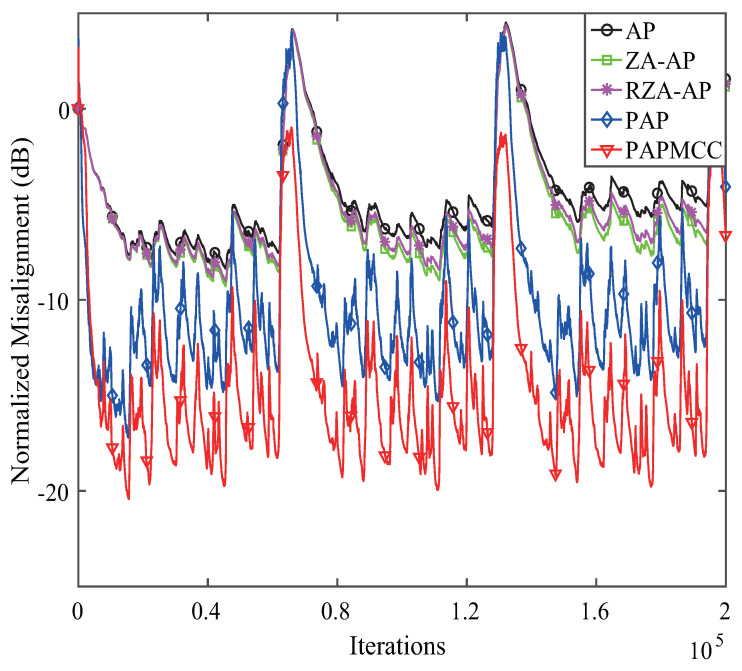
Performance comparisons of the proposed PAPMCC algorithm. Input signal: speech signal.

According to the analysis presented above, we found that the devised PAPMCC algorithm had a lower steady-state MSE when σ and μ were selected. Herein, the estimation behaviors of the PAPMCC was compared with the AP, RZA-AP,ZA-AP, and PAP algorithms. All algorithms were investigated by using WGN, colored noise, and speech signal as input signals, and the sampling frequency for the speech signal was 8 kHz. The used speech signal in this simulation is presented in Figure 6. The performance comparisons of the PAPMCC algorithm with various inputs for network echo channel are presented in Figure 7, Figure 8 and Figure 9, respectively. The PAPMCC algorithm achieved the lowest NM for the sake of comparison with the ZA-AP, AP and RZA-AP algorithms. The PAPMCC algorithm had lower steady-state error while its convergence speed was similar to that of the PAP algorithm. When the input signal was speech signal, the proposed PAPMCC was still better than the related algorithms by considering the convergence and estimation error.

### 4.3. SNR vs. Normalized Misalignment (NM) of the PAPMCC Algorithm

**Figure 10:**
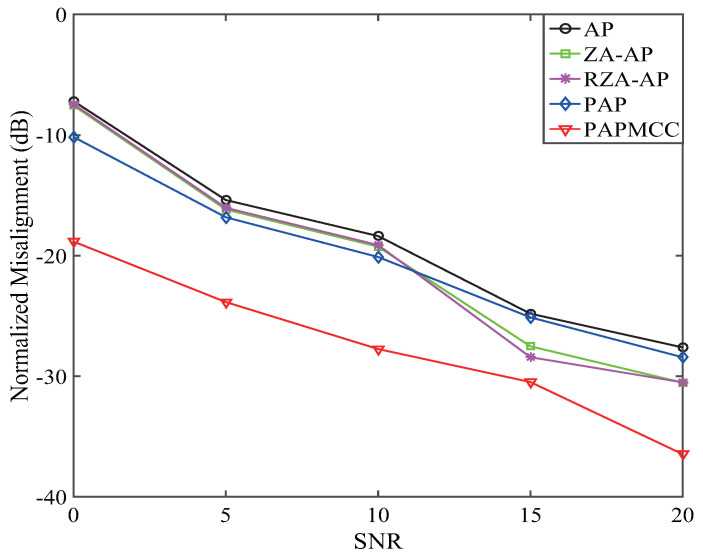
Effects of SNR on the PAPMCC algorithm.

NM versus SNR was used to analyze the performance of the devised PAPMCC under colored input for estimating network echo channel. The performance results of the PAPMCC with various SNRs are presented in Figure 10, which shows that the estimation error decreased as the SNR increased from 0 to 20 dB. Clearly, the steady-state performance of the PAPMCC was significantly better than the related algorithms in low SNR environments.

### 4.4. Performance Comparisons of the Proposed PAPMCC Algorithm with the Conventional Robust AP Algorithms

**Figure 11:**
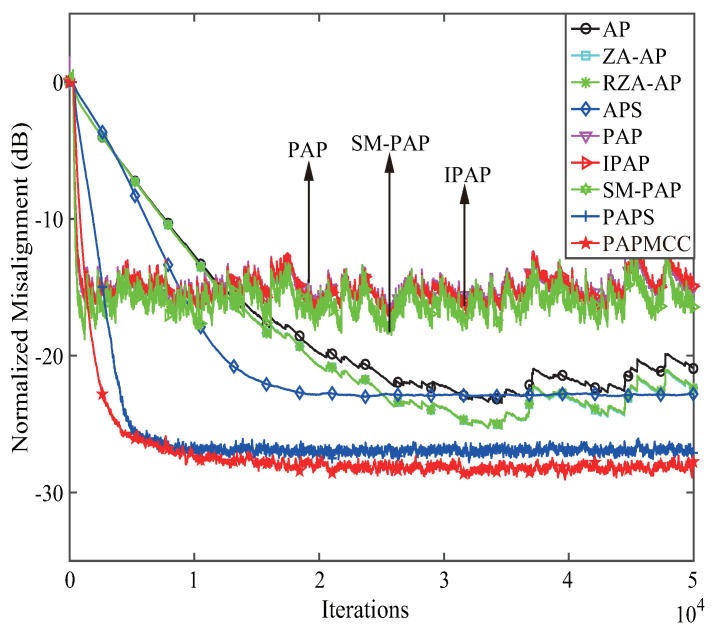
Performance comparisons of the proposed PAPMCC algorithm with the conventional robust AP algorithms. Input signal: colored.

Two conventional robust algorithms were taken into account for comparison to deal with impulsive noise, namely, APS and PAPS algorithms. Herein, the step sizes of the APS and PAPS algorithms were set to 0.005, while the step sizes for the PAP and improved PAP (IPAP) algorithm [49] were set to 0.5, and the bound of set-membership PAP (SM-PAP) algorithm [34] was set to $\sqrt{2\sigma_{r}^{2}}$ where $\sigma_{r}^{2}$ represents the power of the noise. The other parameters were consistent with the previous simulations. The results presented in Figure 11 indicate that the proposed PAPMCC algorithm could still achieve the lowest steady-state error.

## 5. Conclusions

In this paper, the proportionate affine projection maximum correntropy criterion (PAPMCC) has been put forward by the combination of the proportionate and affine projection schemes with the MCC to get a new cost function from the concept of the PAP. The proposed PAPMCC algorithm is carefully derived and investigated via the simulation from various experiments. The results indicate that the PAPMCC algorithm clearly improves the ability of the traditional PAP algorithm under impulsive noise environments. Moreover, compared with the AP, PAP, ZA-AP, RZA-AP, IPAP, APS, PAPS and SM-AP algorithms, the proposed PAPMCC algorithm achieves the lowest NM under three different input signals for estimating network echo channels.

## Figures and Tables

**Table 1:** Computational complexity in each iteration.

Algorithm	Addition	Multiplication	Division
AP	$(2M^{2}+M)K$	$\left(2M^{2}+3M\right)K+M^{2}$	0
ZA-AP	$(2M^{2}+M+1)K$	$\left(2M^{2}+3M+1\right)K+M^{2}$	0
RZA-AP	$(2M^{2}+M+2)K$	$\left(2M^{2}+3M+2\right)K+M^{2}$	*K*
PAP	$2MK^{2}+\left(2M^{2}-M+1\right)K-1$	$2MK^{2}+\left(2M^{2}+3M+1\right)K+M^{2}$	*K*
PAPMCC	$2MK^{2}+\left(2M^{2}-M+1\right)K-1$	$2MK^{2}+\left(2M^{2}+3M+1\right)K+M^{2}+2M$	K+M
